# Revisiting Darwin's comparisons between human and non-human primate facial signals

**DOI:** 10.1017/ehs.2022.26

**Published:** 2022-06-23

**Authors:** Eithne Kavanagh, Clare Kimock, Jamie Whitehouse, Jerome Micheletta, Bridget M. Waller

**Affiliations:** 1 Department of Psychology, Nottingham Trent University, Nottingham, UK; 2 Department of Psychology, University of Portsmouth, Portsmouth, UK

**Keywords:** facial expression, communication, primates, FACS, evolution

## Abstract

Darwin and other pioneering scholars made comparisons between human facial signals and those of non-human primates, suggesting that they share evolutionary history. We now have tools available (the Facial Action Coding System) to make these comparisons anatomically based and standardised, as well as analytical methods to facilitate comparative studies. Here we review the evidence establishing a shared anatomical basis between the facial behaviour of human and non-human primate species, concluding which signals are probably related, and which are not. We then review the evidence for shared function and discuss the implications for understanding human communication. Where differences between humans and other species exist, we explore possible explanations and future directions for enquiry.

## Graphical abstract

**Figure:**
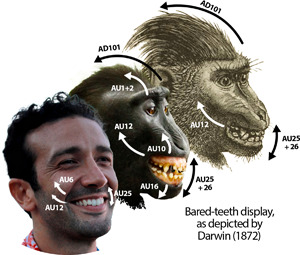

**Social media summary:** Darwin's comparisons between human and animal facial expressions are analysed using anatomically based tools (FACS).

## Introduction

Charles Darwin pioneered the idea that facial displays show continuity across species in *The Expression of Emotion in Man and Animals* (Darwin, 1872). Darwin drew comparisons between specific facial behaviours in humans and non-human animals and argued that they were probably underpinned by the same emotional feeling states. He stated that ‘the expressions and gestures involuntarily used by man and the lower animals, [are] under the influence of various emotions and sensations’. Hence, facial behaviours have become commonly termed ‘facial expressions’. His focus was on providing evidence for continuity of mind and psychological processes (in contrast to form only) across species, which at the time was a controversial proposal. This approach has left a legacy which persists to this day and has paved the way for a better understanding of the phylogenetic origins of human facial behaviour. However, it could be argued that the search for continuity of emotional experience across species has superseded the question of whether there is continuity of the morphological facial configurations themselves. For example, Kret and colleagues discussed whether human emotional behaviours have counterparts in other species, using similarity of behaviours and behavioural context as potential evidence of homology (Kret et al., 2020). Yet if the link between emotion and behaviour is less clear and consistent than Darwin assumed (and we, as well as others, argue that it is: Barrett et al., 2019; Fridlund, 1994), then when considering facial behaviour in particular, such an approach may misidentify phylogenetic links between facial configurations across species. It may also assume that there is homology of emotional experience when behaviours are similar across species, which might not always be the case.

Darwin appeared comfortable with the notion that some behaviours such as facial expression were direct expressions of internal states. Ekman's Basic Emotions Theory (Ekman, 1992) has since solidified the widespread view that facial expressions are essentially synonymous with emotion. This dominant view has been challenged by scholars such as Alan Fridlund, who view facial displays as dynamic social tools used to communicate motivations or intentions (Fridlund, 1994). Fridlund's Behavioural Ecology View of facial behaviour does not exclude the possibility that emotions contribute to such motivations or intentions, but offers an alternative approach that refocuses on external behaviour and social interaction rather than internal states. Moreover, large-scale meta-analyses have found a more complex link between emotion and facial behaviour (Barrett et al., 2019), so the fundamental tenets of Darwin's framework are now more difficult to accept. It may be more fruitful to start with the form of facial configurations across species regardless of what emotion they are thought to express, if any, in order to identify phylogenetically related behaviours (Waller et al., 2020). By focusing on the anatomical structure of these configurations (an approach taken by such early scholars as van Hooff and Andrew; e.g. Andrew, 1963; Van Hooff, 1972), we can examine potentially homologous facial displays and thereby try to understand the continuity of form and function. Evidence for shared anatomical structure may also provide better insight into the phylogenetic path that facial displays have taken over the course of evolution.

As Darwin's (1872) primary aim was to compare the expression of *emotions* in humans and other animals, sometimes across modalities, he only made a few direct comparisons between the morphology of human facial expressions and those of other animals. Darwin's collection of observations was also gathered from isolated case studies of animals, and his focus on the expression of emotions may have missed opportunities to identify facial expressions that are similar in appearance but occur in different contexts in humans and other animals. One way to examine similarity of facial expressions across species is by using FACS (Facial Action Coding Systems). The original FACS (Ekman et al., 2002; Ekman & Friesen, 1978) was created for use with humans and was an attempt to standardise descriptions of facial behaviour. Prior to this, scientific approaches were hampered by lack of a common language with which to describe the complex and often subtle facial movements that form part of human communication. The FACS was based on earlier anatomical work (Hjortsjo, 1970), which described individual facial movements in terms of changes to the underlying facial musculature. The system comprises codes for individual muscles movements, termed action units (AUs) or action descriptors (ADs), which are more global head/eye movements, etc. The system has since been modified for use with a range of non-human animal species (for a summary see Waller et al., 2020), which allows comparisons between species to be made based on morphological similarity. Here, we revisit the comparisons made by Darwin (and other scholars) in their pioneering work and, using FACS, review whether evidence for homology has accumulated since the comparisons were first made.

## Darwin's observations on facial displays across species

### Darwin and smiling and laughing

**Figure 1.**
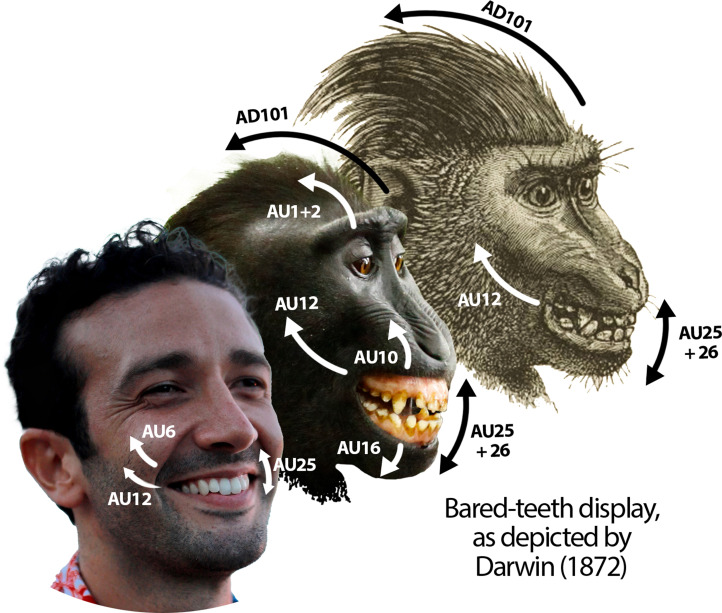
Comparison of human smile, bared-teeth display in a crested macaque (*Macaca nigra*) and the bared-teeth displayed depicted by Darwin (1872). AU1 + 2: inner and outer brow raisers; AU6, cheek raiser; AU10, upper lip raisers; AU12, lip corner puller; AU16, lower lip depressor; AU25, lips parted; AU26, jaw drop; AD101, scalp retractor. Human photo credit to Roman Shilin/Unsplash.com

Darwin (1872) made a number of observations on the similarities between human smiling and laughter and facial displays found in chimpanzees (*Pan troglodytes*), orangutans (*Pongo spp*), monkeys (*Cebus azarce*, *Cebus albifrons*, *Cynocephalus anubis*, *Macaca niger* and *Macaca sylvanus*) and dogs (*Canis lupus familiaris*). Darwin noted that chimpanzees ‘laugh’ when pleased, and while doing so ‘the lips are protruded’ (p. 131). When tickled, they laugh and ‘the corners of the mouth are then drawn backwards, and this sometimes causes the lower eyelids to be slightly wrinkled’ (p. 131). Likewise, he reported that chimpanzees and young orangutans ‘grin and make a chuckling sound’ when tickled, and afterwards an expression which ‘may be called a smile’ passes over their faces (p. 132), thus an ‘expression of satisfaction, partaking of the nature of an incipient smile, and resembling that often seen on the face of man, could be plainly perceived in this animal’ (p. 132). He noted in monkey species (e.g. *Cebinae*, *Macaca*) that during certain positive contexts they emit various types of vocalisations, and ‘draw back the corners of [their] mouth[s], apparently through the contraction of the same muscles as with us’ (p. 132). He added that in crested macaques (*Macaca nigra*), the mouth corners are also drawn upwards, exposing the teeth (see Figure 1), and in both Barbary (*Macaca sylvana)* and crested macaques, the lower eyelids become wrinkled. He commented that such wrinkling in monkeys is ‘so characteristic of our own laughter’ (p. 131). He also tentatively compared dogs grinning when expressing fondness with that of a human smile, ‘but if it had been really a smile, we should see a similar, though more pronounced, movement of the lips and ears, when dogs utter their bark of joy’ (p. 120).

**Figure 2.**
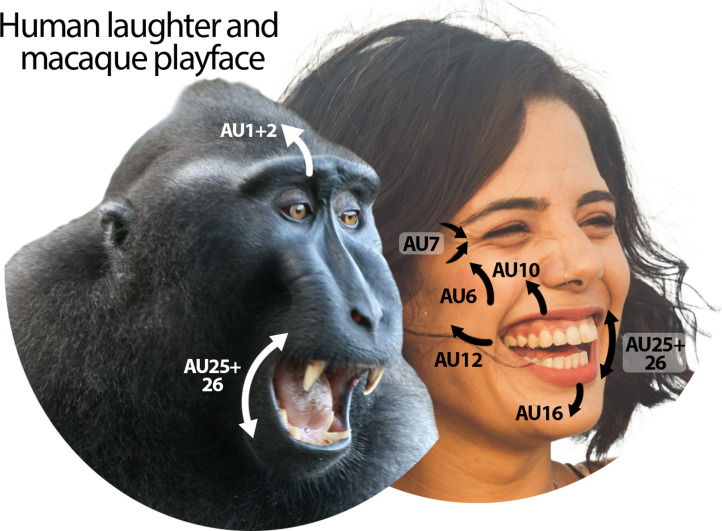
Comparison of human laugher and non-human primate playface (crested macaque, *Macaca nigra*). AU1 + 2, Inner and outer brow raisers; AU6, cheek raiser; AU7, lid tightener; AU10, upper lip raiser; AU12, lip corner puller; AU16, lower lip depressor; AU25, lips parted; AU26, jaw drop. Human photo credit to Chermiti Mohamed/Unsplash.com.

Darwin's observations have received empirical support to an extent. Smiling and laughing have been by far the most-studied facial displays, and many of his observations are supported by empirical data. Evidence suggests that the two have separate phylogenetic origins. Early scholars initially assumed that smiling was the diminutive of laughter and that both displays derived from the relaxed open mouth display (ROM), also known as ‘play face’ (Bolwig, 1964; Hayworth, 1928; Van Hooff, 1972). The ROM is thought to be a ritualised display emerging from preparation for mock biting during play, and is commonplace among predatorial species (e.g. canids, Fox, 1970); brown bears, *Ursus arctos*, Egbert et al., 1976), see Figure 2.

As Preuschoft (2000) states, ‘the play face is the ancient heritage of all primates, potentially dating back from cretaceous times’ (p. 266). Its function can be classed as essentially affiliative as it communicates a lack of intent to harm in earnest, and so mirrors the generally socio-positive functions of smiling or laughing. Many scholars therefore suggested that the relaxed open mouth display became ‘fully ritualised as smile or laughter’ in humans (Bolwig, 1964: 183) owing to their contextual and morphological similarities. However, it is now more widely acknowledged that smiling and laughter most likely had separate origins, and only later did they converge contextually and morphologically to some extent (Preuschoft & van Hooff, 1995; Van Hooff, 1972).

The smile is now widely thought to have its origins in the silent bared teeth display (SBT; Preuschoft & van Hooff, 1995; Van Hooff, 1972). This appears in many primate species, and in most it signals appeasement and/or fear. Its direction is typically asymmetrical, produced by subordinate individuals towards dominant partners, signalling submission. However, in species with more relaxed dominance hierarchies, such as tonkean (*Macaca tonkeana*) and lion-tailed (*Macaca silenus*) macaques, it is produced symmetrically between individuals and may be a signal of reassurance (the Power Asymmetry Hypothesis; Preuschoft & van Hooff, 1997). Importantly, it is in these same species that the ROM display is not restricted to the play context, but is also produced in friendly interactions. In other words, the contexts of the ROM display and the SBT display overlap in tolerant primate species, just as the contexts of smiling and laughter do in humans. Additionally, these more tolerant species produce ROM displays that are morphologically more similar to the SBT display than those in despotic species, as they bare the upper teeth row (Preuschoft & van Hooff, 1995; Van Hooff, 1972). Again, this mirrors the blending of human displays of smiling and laughing. As such, according to the Power Asymmetry Hypothesis, the relaxation of dominance hierarchies coincided with the blending of the ROM and SBT display, both morphologically and contextually. Similarly, given the supposed egalitarian social structures of ancestral humans (Boehm, 1999), it is thought that this factor also coincided with similar blending of smiling and laughter, but that they have separate phylogenetic roots with the smile emerging from the SBT display, and laughter emerging from the ROM (Preuschoft & van Hooff, 1997).

Davila-Ross and Dezecache (2021) recently challenged the Power Asymmetry Hypothesis. In contrast, they suggested that the human smile and laugh both evolved within the context of play and have a shared ancestry, referring to this as the ‘Complexity and Phylogenetic Continuity’ hypothesis. A key factor in support of their hypothesis is that chimpanzees have been shown to bare their teeth along with ROM, mirroring the baring of teeth that accompanies laughter in humans. As such they argued that the most parsimonious explanation is that smiling and laughter emerged from a single graded SBT–ROM display in our last common ancestor. It is certainly a plausible suggestion that the displays merged before the emergence of modern humans, although an explanation is still needed for the relationship between power asymmetry and blending of the two displays in more phylogenetically distant species such as macaques (Preuschoft & van Hooff, 1997). An explanation is also needed for the subtle but important differences in usage between smiles and laughs in humans. Davila-Ross and Dezecache (2021) provide evidence that the addition of bared teeth to a laugh or a play face appears not to substantially alter its meaning in chimpanzees, and that laughing is almost invariably accompanied by a smile in humans. However, the reverse does not appear to be true. There are narrow contexts in which a smile is appropriate where a laugh is not (e.g. greeting; Eibl-Eibesfeldt, 1972), just as the SBT face is used in narrow contexts in chimpanzees where the play face is not (e.g. signalling submission; Van Hooff, 1972). The Power Asymmetry Hypothesis provides a plausible explanation for these phenomena where the Complexity and Phylogenetic Continuity hypothesis does not.

Smiles in humans may also still perform some of the hierarchy-management functions as their proposed predecessor, the SBT. The direction of deliberate smiles, but not spontaneous smiles, is associated with hierarchical relationships in humans (Mehu & Dunbar, 2008). Smiling in humans is also associated with decreased physical dominance (Kraus & Chen, 2013; Witkower et al., 2020), but with increased prestige (an alternative, human-specific strategy of obtaining higher status; Cheng et al., 2013; Witkower et al., 2020). Interestingly, in an ecological analysis of school children, Sarra and Otta (2001) found that a broad smile and laughter were associated with playfulness–mock aggression, while a closed and upper smile was associated with friendliness–appeasement. Hierarchical differences also appear to explain the gender stereotype that women smile more. Women are perceived as lower in dominance and those low in dominance are expected to smile more; this fully mediates the gender stereotype (Hess et al., 2005).

### Darwin and anger/concentration/determination

Darwin (1872) identified similarities in faces of ‘concentration’ in orangutans and humans. He compared humans’ tendency to close the lips firmly when performing difficult and precise tasks with a similar action he observed in a young orangutan; ‘The poor little creature was sick, and was amusing itself by trying to kill the flies on the window-panes with its knuckles; this was difficult as the flies buzzed about, and at each attempt the lips were firmly compressed, and at the same time slightly protruded’ (p. 140). It is conceivable that facial displays of concentration would be selected for across primates, as they could benefit the producer by preventing the interruption of important survival tasks. For tasks with high cognitive demands, a lower level of arousal is optimal (Yerkes & Dodson, 1908), so concentration displays may signal to conspecifics to not interact with them as they are approaching their optimal or maximum capacity. Keltner and Shiota (2003) suggested such displays could communicate ‘I've got all the information I can handle right now – don't send any more yet’ (p. 89). A receiver may only be likely to comply with such a request, however, if there is a benefit to them, such as in a cooperative environment or when failing to comply would result in aggression. This is, however, an untested hypothesis, and studies measuring recipient responses to concentration displays across species would be valuable to investigate this possibility.

While Darwin made observations on a ‘concentration face’, some have suggested that this may be closely related to the ‘anger face’ owing to their morphological similarities (Bolwig, 1964; Harmon-Jones et al., 2011), which has been more widely studied. The so-called ‘anger face’ in humans comprises furrowed brows, raised eyelids, narrowed eyes, raised chin and pressed lips (AU4 + AU5 + AU7 + AU17 + AU24; Ekman & Friesen, 1978). Bolwig (1964) suggested that a similar facial expression is produced during concentration and anger, in both humans and in other primates. He described ‘a frowning and a tightening and inwards bending of the lips’ (p. 188) associated with concentration, or with anger, in humans, apes and monkeys. Some findings suggest that concentration could be an under-appreciated context of the ‘anger’ facial configuration in humans. Rozin and Cohen (2003) found that a high frequency of facial expressions was interpreted by a sample of college students as ‘thinking–concentration’. Although these students were not FACS trained, they often interpreted narrowing of the eyes and a lowering and coming together of the eyebrows as indicative of concentration, which could be produced by AUs 7 and 4. Harmon-Jones et al. (2011) found that the prototypical FACS configuration of anger is perceived similarly to determination – a related concept. Darwin (1872) also remarked that ‘the firm closure of the mouth tends to give an expression of determination or decision to the countenance’ (p. 233), which suggests the use of lip press (AU24). Preventing obstruction of the producer's goals without an aggressive outcome could be the shared function of this display in both ‘anger’ and ‘concentration’ contexts. Indeed, this is consistent with Waller et al.'s (2016) finding that a crested macaque did not associate bared teeth face, threat or scream face with a conflict outcome, indicating that facial expression in general may reduce the likelihood of social conflict.

There is some convincing evidence that the human ‘anger face’ (and thus the ‘concentration face’) could have a non-human primate counterpart. Preuschoft (2000) compared the compressed lips component of this display with the ‘bulging lip face’ of chimpanzees which is produced when bluffing and charging. Bolwig (1964) also suggested that anger is expressed similarly in humans and apes, which he described as ‘frowning, stiffening of the gaze and tightening of the lips while the mouth is pulled downward’ (p. 186), while Andrew (1963) suggested that humans and chimpanzees both draw the eyebrows together during threat. While Parr et al. (2007) did not have a sufficient sample size to identify bulging lip face as a prototypical display in chimpanzees, they found that the chin raise (AU17) and lip pressor (AU24) are anatomical equivalents produced both in the anger face and the bulging lip face, suggesting that both displays may be homologues. Frowning (AU4) is not present in chimpanzees (ChimpFACS; Vick et al., 2007) and so could not accompany bulging lip face as Bolwig (1964) and Andrew (1963) indicated. Preuschoft (2000) noted that the ‘tense mouth face’ which precedes attack in many macaque and baboon species (and was often accompanied by lowered brows) was probably the precursor to the bulging lip face (van Hooff, 1967), which would suggest a homology going back at least 5 million years. The contexts of these displays are comparable in their aggressive connotations. However, AU24 (lip press) has also not been identified in macaque or baboon species (although a FACS for baboons has not yet been developed), suggesting no evidence that the tense mouth face preceded the bulging lip face. It is probable that the tense mouth face described by van Hooff (1967) is produced by different AUs (potentially AU8 – ‘lips towards each other’; he described the lips as ‘closed or almost closed and … may be drawn inward’, p. 18), and is therefore not a good candidate as a homologous precursor to bulging lip face or human anger face. Together, preliminary FACS data indicates that a potential homology between human anger/concentration face and chimpanzee bulging lip face is worth pursuing, but that the tense mouth face may be unrelated, pending the development of a baboon FACS.

However, Waller et al. (2014) examined the use of chin raise and lip press (AU17 + AU24), both components of ‘anger/concentration/determination’ and ‘bulging lip’ face, in children and chimpanzees. They found that production of these AUs predicted persistence in an impossible task in children, but not chimpanzees. This is suggestive of a lack of continuity between the use of this expression in this context, although the presence of a conspecific (the experimenter) for the children but not the chimpanzees (although there was a human experimenter present) could go some way to explain differences in the production of facial expression. It could be worth testing whether chimpanzees produce ‘concentration displays’ in the presence of conspecifics, in particular a lower-ranking conspecific, for whom the prevention of aggression would be most beneficial. There are no comparable studies using FACS with the other species identified as producing concentration displays by Darwin (1872) and Bolwig (1964; orangutans, gorillas, macaques and patas monkeys).

### Darwin and the pout face

**Figure 3.**
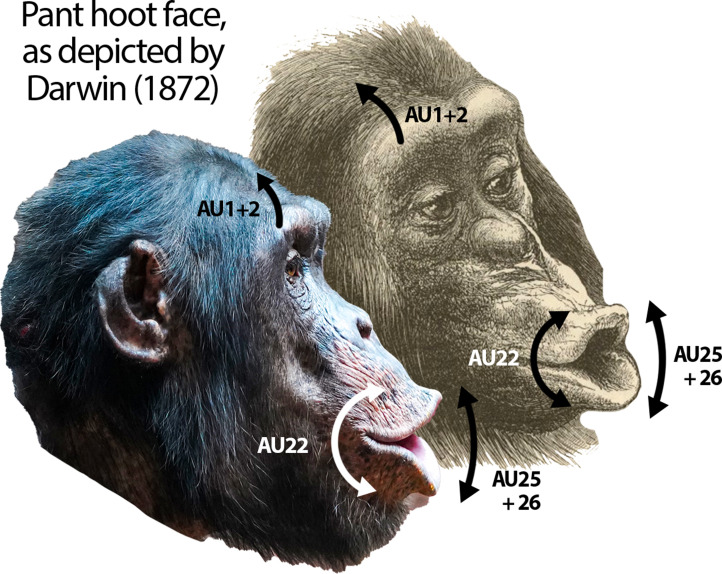
Chimpanzee pant hoot, with similar expression depicted by Darwin (1872: 141): ‘Chimpanzee disappointed and sulky’. AU1 + 2, Inner and outer brow raisers; AU22, lip funneler; AU25, lips part. Photo credit to glog192/Shutterstock.com.

Darwin (1872) compared the protrusion of the lips in chimpanzees and orangutans ‘when slightly angered, sulky, or disappointed, … [or] when alarmed at anything’ with that of ‘sulky children’ (see Figure 3). Bolwig (1964) later also noted that ‘the chimpanzee, when unhappy after being scolded’, seems to produce a ‘protrusion of the lips’ (p. 185), which may indicate a mixture of fear and sadness. These descriptions seem to match what is termed the ‘pout face’ in chimpanzees and orangutans. This was identified as a distinct facial configuration in ChimpFACS and consists of raised chin, lips funnelled and lips parted (AU17 + AU22 + AU25; Parr et al., 2007). Parr et al. (2007) suggested that chimpanzee pouts may represent a need for contact, reassurance and physical affinity, given the approach-related contexts in which they are produced (e.g. embraces). The same action units are present in OrangFACS (Caeiro et al., 2013), and the orangutan pout face which matches this description is often produced as a submissive request for tolerance or appeasement (Liebal et al., 2006; Rijksen, 1978). Using a naturalistic approach, Hardecker et al. (2021) identified lowering the brow and pouting the lips as indicative of sulking in young children, although they did not use FACS. They suggested that sulking could be a resource control strategy by less powerful individuals, by requesting bond repair via threats of withdrawing affection. Gaspar (2006) asserted that pout face is a good potential candidate for a homology with a bonding-promoting function, although it is also worth exploring the role of relative dominance status in its function, as there are interesting similarities in production context across species with regards to the submissive status of the producer. It could be a valuable avenue for future research to investigate homology of the pout face, by identifying the relative status of the producer and receiver, and whether the signal elicits affection or resource provision from the receiver.

Andrew (1963) noted that contraction of the orbicularis oris which produces lip funneler (AU 22) is necessary for many vocalisations: a roaring lion, a howling dog, a lowing deer. He suggested that the production of this visual signal alone announces the onset of vocalisations in lemurs, Ceboidea, Cercopithecoidea and Hominoidea, and compares this with the protrusion of the lower lip when a human child is about to cry. This appears to be a plausible suggestion for the origin of the pout face, and the lip funneler appears in FACS for humans, chimpanzees, gibbons, orangutans and horses. However, it does not appear in FACS for dogs, cats or macaques, although lip pucker (AU 18) does appear, which also involves contraction of the orbicularis oris.

While Darwin appeared to be addressing the pout face, the image he presented of a ‘chimpanzee disappointed and sulky’ appears to match the facial expression associated with pant-hoots (Darwin, 1872; p. 139; see Figure 3). When producing ‘pant-hoot’ vocalisations, chimpanzees produce a facial expression that shares some morphological similarities with the human pout face (Vick et al., 2007). The ‘pant hoot face’ comprises raised eyebrows (AU1 + AU2), an open mouth (AU25 + AU26) and protruded lips (AU22; Vick et al., 2007). Although Darwin (1872) seems to associate this expression with pouting, contemporary researchers tend to agree that there is no human morphological equivalent to the pant hoot face (Parr and Waller, 2006; Parr et al., 2007).

## Other facial displays with potential cross-species continuity

While Darwin (1872) made the above three direct comparisons between human and non-human animal facial expression, a number of scholars have made other credible comparisons, such as the ‘scream face’ and the ‘disgust/rain face’.

### Scream face

**Figure 4.**
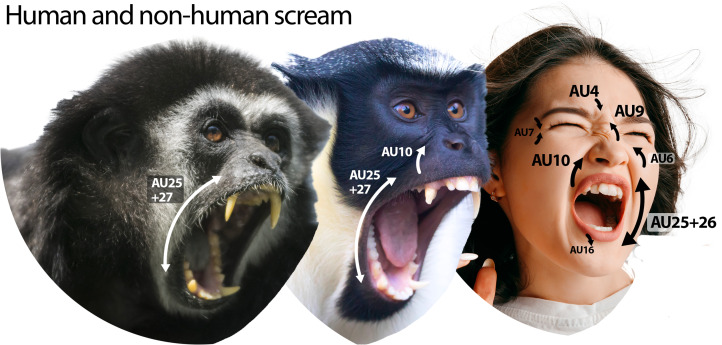
Human and non-human primate scream displays. Left, Lar gibbon (*Hylobates lar*); middle, Diana monkey (*Cercopithecus diana*). AU4: Brow lowerer; AU6, cheek raiser; AU7, lid tightener; AU9, nose wrinkler; AU10, upper lip raiser; AU16, lower lip depressor; AU25, lips parted; AU26, jaw drop; AU27, mouth stretch. For FACS coding of the Diana monkey (middle), action units on this figure are approximations because an established FACS coding system does not currently exist for this species. Photo credits to Stefan Wolny/Shutterstock.com, Jiri Fejklon/Shutterstock.com and Master1305/Shutterstock.com.

Parr et al. (2007) compared the ‘scream face’ in chimpanzees with that of humans and suggested that they are potential homologues. This configuration in chimpanzees shares five of the eight action unit involved in the human configuration (see Figure 4). Screams are also produced in similar contexts, generally in response to threats or other sources of distress (Kersken, 2012; Slocombe & Zuberbühler, 2010). A similar scream face is probably produced in multiple species, such as barbary macaques (Hesler & Fischer, 2007), capuchin monkeys (*Cebus apella*; Weigel, 1979) and gorillas (*Gorilla gorilla gorilla*; Brann, 1999), although a comparable FACS approach has not yet been employed across species. Chimpanzees produce different scream ‘types’, in that their acoustic properties differ depending on the social role of the producer and audience composition (Slocombe & Zuberbühler, 2005, 2007), but whether such differences are reflected in the appearance of the associated scream faces is unknown (Parr et al., 2007).

### Disgust/rain face

The prototypical human ‘disgust’ face comprises brow furrow, nose wrinkle, upper lip raise, tongue protrusion and jaw drop (AU4 + AU9 + AU10 + AU19 + AU26). Steiner and Glaser (1984) found that neonates of humans and multiple primate species display a ‘deterred’ facial expressions to a bitter taste, which includes tongue protrusion, depressed mouth angles and gaping mouth (and additionally nose wrinkle in human infants and adult apes; Steiner et al., 2001). Although the gaping mouth and nose wrinkle components could appear to suggest some continuity with the ‘disgust’ face, Kunz at al. (2013) found that the upper lip raise (AU10) most strongly encodes ‘disgust’ in humans, and this does not appear in the ‘deterred’ facial expression. However, de Waal (2019), however, described a ‘rain face’, which he observed chimpanzees producing during heavy downpours, which includes pulling the upper lip close to the nose, showing the teeth, sticking the lower lip out, and semi-closing the eyes. It is interesting to note that one salient component of the ‘disgust’ face, nose wrinkle (AU9), does not appear in the rain face, and chimpanzees appear to produce nose wrinkle rather infrequently (Parr et al., 2007). While it could be that the facial movements observed across primate species have a protective function to aversive stimuli, it could be that the combination of the nose wrinkle and upper lip raise in humans exaggerates the ‘facial compression’ effect to increase salience of the signal. This would highlight how facial movements selected for non-social purposes such as individual regulation or sensory management could be co-opted and adapted for a social function. The ‘disgust face’ in humans may function to deter ingestion of pathogens in observers, which benefits the producer as well by reducing exposure to disease from conspecifics (Chapman & Anderson, 2012). It is also produced in response to social rule violations, and may function to regulate the social behaviour of others, or to communication moral motivation to others (Chapman & Anderson, 2013; Kupfer & Giner-Sorolla, 2017), although there are debates as to whether the concept of ‘moral disgust’ has a primarily cultural or biological basis (Rozin & Haidt, 2013).

## Potentially species-unique facial expressions

There are a number of human facial expressions which do not appear to have a counterpart in non-human primates, and similarly, some facial expressions that are produced by non-human primates appear not to be produced by humans. We outline a number of these described by Darwin (1872).

### Darwin and human crying/‘sad face’

It is possible that those human facial expressions without a clear counterpart in other animals tend to be those which expose some form of vulnerability in the producer. Waller et al. (2013) suggested that such displays can only be functional if receivers are likely to provide assistance. One such display is crying, which may function as a request for assistance or communication of sadness (Ekman, 1992; Fridlund, 1994). Darwin (1872) identified that ‘sobbing seems to be peculiar to the human species’ (p. 156) and indeed no other species has since been reliably documented to shed tears. With regards the accompanying facial configuration, he asserted that, as infants cry, ‘their eyes are firmly closed, so that the skin round them is wrinkled, and the forehead contracted into a frown. The mouth is widely opened with the lips retracted in a peculiar manner, which causes it to assume a squarish form; the gums being more or less exposed’ (p. 147). He drew particular attention to the oblique shape of the brows (which he termed ‘grief muscles’) and the drawing down of the corners of the mouth, which he suggested occurs during times of great distress in humans. He suggested that ‘this produces peculiarly-formed wrinkles on the forehead, which are very different from those of a simple frown’ (p. 177). His descriptions are in line with the prototypical FACS configuration of ‘sadness’ (AU1 + AU4 + AU15; Ekman et al., 2002). While Darwin (1872) did not make comparisons between these facial movements in humans and other animals, Bolwig (1964) claimed that sadness is expressed with a similar facial expression in monkeys and humans and in apes in higher intensities. It is unlikely that the down-turned mouth component has continuity across species as this action unit (AU15) does not appear in any FACS outside of humans. However, Bolwig's description of ‘slanted eyebrows’ with lips ‘slightly pouched’ is reminiscent of the ‘tense mouth’ display described by Preuschoft (2000), who indicated that this was produced not only in threat but also in a fear context. Both authors also included baboons in their observations. It is possible that ‘slanted eyebrows’ (i.e. AU1 + AU4) could function as a request for assistance, although more investigation of the ‘tense mouth’ display using FACS is needed. Interestingly, Waller et al. (2013) found that shelter dogs who produce slanted eyebrows (AU101) more frequently were more likely to be adopted. They suggested that this could be due to an enhanced appearance of paedomorphism and perceived vulnerability, which could have elicited a caretaking response from prospective human owners, and seems to have coevolved with changes to dog facial musculature (Kaminski et al., 2019). This is, however, unlikely to have shared ancestry with the human ‘sad face’, as there is no evidence of its continuity in any phylogenetically intermediate species. It is more likely that it evolved during selective breeding to appeal to human caretakers.

### Darwin and the human fear face

The prototypical facial expression of fear in humans comprises raised eyebrows and wide eyes and mouth (AU1, AU2, AU4, AU5, AU20 and AU25; Ekman et al., 2002), which reflects Darwin's (1872) description of such an expression whereby ‘the eyes and mouth are widely opened, and the eyebrows raised’ (p. 289). Again, while Darwin (1872) did not draw any clear comparisons with this configuration in other animals, Bolwig (1964) suggested that faces of frightened humans are similar to those of frightened monkeys (patas monkeys and baboons), chimpanzees and dogs. While there is no FACS yet developed for patas monkeys or baboons, we can assume that the comparison with chimpanzees and dogs is unfounded as there is no AU4, AU5 or AU20 present in the FACs for either species, or additionally AU1 or AU2 in dogs.

### Darwin and ear movements

Darwin (1872) noted that many non-human animals, including several species of non-human primates, incorporate ear movements into their facial expressions. Macaques are one non-human primate group in which ear movements are important components of facial expressions (e.g. Parr et al., 2010; Partan, 2002). MaqFACS includes several codes to describe ear movements (e.g. EAU1, ears forward; EAU2, ear elevator; EAU3, ear flattener; EAD, general ear movement; Parr et al., 2010). Other non-human primate taxa, such as chimpanzees, also incorporate ear movements into their facial expressions (Vick et al., 2007), but MaqFACS is so far the only non-human primate FACS coding system that includes separate codes for ear movements. Cats, dogs and horses also have mobile ears, and DogFACS (Waller et al., 2013), CatFACS (Caeiro et al., 2017) and EquiFACS (Wathan et al., 2015) include codes to describe such movements.

### Darwin and lip-smacking face

**Figure 5.**
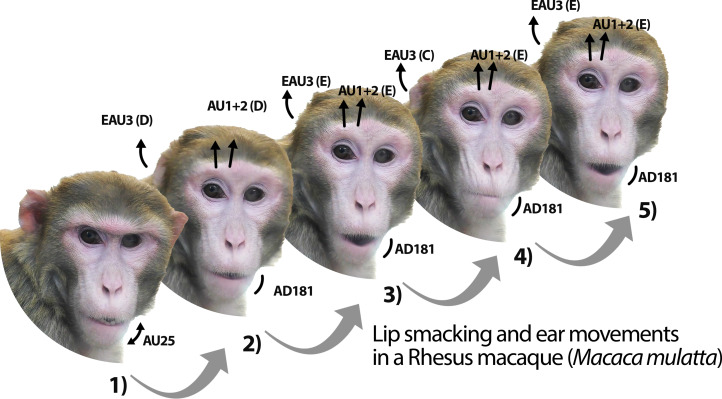
Lip smacking and ear movement sequence in a Rhesus macaque across time. (1) Neutral face with lips parted (AU25); (2) ears begin to flatten (EAU3), inner and outer brows raise (AU1 + 2), and rhythmic smacking of the lips occurs (lip smacking, AU181). For the remainder of the display, there is rhythmic movement of the ears and lips. In FACS notation, letters denote the intensity of the movement wherever relevant (low intensity A, to high intensity E).

Darwin (1872) described a facial expression in which monkeys ‘retract their ears, show their teeth, and jabber’ (p. 114) and another in which a monkey ‘rapidly moved its lower jaw and lips in a spasmodic matter, the teeth being exposed’ (p. 132). Although Darwin did not name these expressions, these descriptions appear to match what is now commonly referred to as lip-smacking. Similar to the ‘pant-hoot’ face described above, the ‘lip-smacking’ face is part of a signal involving both facial movement and sounds (Micheletta et al., 2013). The primate ‘lip smacking face’ involves lowering and raising of the mandible (AU26/AU27), opening and closing of the lips and/or mouth (AU25), and protrusion and retraction of the tongue (AD19; van Hooff, 1967). This expression is sometimes also accompanied by raised eyebrows (AU1 + AU2) and retracted ears (MaqFACS EAU3, see Figure 5). Lip-smacking has been described in multiple species of African and Asian monkeys, chimpanzees and human infants, although the exact configuration of the expression varies (e.g. chimpanzees, Nishida et al., 1999; multiple species of macaques, Preuschoft & van Hooff, 1995; geladas, Bergman, 2013; mandrills, baboons, patas monkeys, and mangabeys, van Hooff, 1967; human infants, Steiner et al., 2001). There is a MaqFACS code specifically for lip smacking (AD 181, lip smacking: Parr et al., 2010), but ChimpFACS (Vick et al., 2007) does not include a separate code. Although the series of movements that comprise lip-smacking seems to be unique to certain species of non-human primates, one of the components of lip smacking, the lip pucker, is produced by both humans and non-human primates (AU 18 FACS; AU18i and AU18ii, MaqFACS; Parr et al., 2010). Many species that lip smack (e.g. geladas, baboons and mangabeys) do not yet have an associated FACS so it is difficult to make cross-species morphological comparisons among the configurations of lip-smacking. This facial expression is generally produced during or prior to affiliative interactions such as grooming (chimpanzees, Fedurek et al., 2015; rhesus macaques, Maestripieri & Wallen, 1997) and in interactions between mothers and infants (Maestripieri & Wallen, 1997). In infants of both humans and non-human apes, lip smacking has also been documented in response to sweet-tasting foods (Steiner et al., 2001; Steiner & Glaser, 1984). Therefore, while there is no evidence of a direct counterpart in humans, it is possible that future data may identify continuity with this expression in humans.

### Darwin and open-mouthed threat face/staring open-mouth face

Darwin (1872) documented the use of an open-mouth facial expression in aggressive interactions between non-human primates. Describing a Barbary macaque, he writes that ‘the [individual's] mouth was open much more widely, the canine teeth were more fully exposed’ (p. 133). The open-mouth threat includes raised eyebrows (AU1 + AU2), raised upper eyelids (AU5), retracted (and/or flattened) ears (MaqFACS EAU2/EAU3) and a lowered jaw and open mouth (AU25 + 26) with protruded corners and tightened lips (AU22) (Parr & Heintz, 2009; Preuschoft, 2000; van Hooff, 1967). The ‘open-mouthed threat face’ (or ‘staring open-mouth face’) is displayed by a range of non-human primate species (e.g. macaques, mangabeys, chimpanzees, guenons and colobus monkeys), but the configuration of this facial expression varies across species (van Hooff, 1967). For example, in rhesus macaques (*Macaca mulatta*), this expression includes a jaw thrust (AD29 in humans) and the mouth is open such that the lower teeth are visible, but the upper canines are not (Hinde & Rowell, 1962). This facial expression is produced in agonistic (conflict) contexts and is typically produced by the aggressor and/or an individual of higher rank than the receiver (Hinde & Rowell, 1962).

## Conclusion

In sum, some of Darwin's (1872) original comparisons between human facial displays and those of other primates have held up to empirical scrutiny and others have not. Smiling and laughing comparisons with other species have by far received the most scientific attention and there is good evidence to suggest that these human displays are rooted in ancestral displays and exhibit continuity across species. Evidence for cross-species continuity of anger/concentration displays and ‘sulky’ displays is less clear, but still these observations have led to promising lines of research. It is also important to acknowledge that there may be morphological variations of all of the facial displays discussed in the current paper. For instance, recent evidence identified different smile categories and functions, depending on the accompaniment of other action units, with the addition of eyebrow raising signifying ‘reward’ smiles, lip pressing signifying ‘affiliative smiles’ and nose wrinkling and lip raising indicating ‘dominance’ smiles (Martin et al., 2017; Rychlowska et al., 2017). Similarly, different forms of SBT have been demonstrated to perform different functions in chimpanzees (Van Hooff, 1972) and crested macaques (Clark et al., 2020). More research is needed on these kinds of variations across all facial displays and how they affect social outcomes. This, along with widespread phylogenetic comparisons, is likely to be illuminating in identifying truly homologous facial behaviour.

On the whole Darwin (1872) made a pivotal contribution to our understanding of the evolution of facial expressivity by describing and comparing facial expressions across species. His comparisons are limited, however, by a focus on emotional expressions of assumed internal states. In order to make more robust evolutionary inferences about the evolution of human facial expressivity, we need more naturalistic studies, especially in humans, to investigate the social function of facial expressivity. We also need to employ anatomically based systems to describe facial movement, like FACS (e.g. Human FACS, Ekman et al., 2002; ChimpFACS, Vick et al., 2007; MaqFACS, Parr et al., 2010; GibbonFACS, Waller et al., 2012; OrangFACS, Caeiro et al., 2013; DogFACS, Waller et al. 2013; CatFACS, Caiero et al. 2017; EquiFacs, Waltham et al. 2015), to compare the morphology of facial expressions across species. The development of FACS for more non-human primate taxa, such as baboons, geladas and monkeys from Central and South America, and for more non-primate animal taxa, will facilitate such comparisons. Our understanding of the evolution of facial expressions has increased significantly since the publication of Darwin's (1872) work, but more research into the form and function of facial expressions is still needed.

## Research transparency and reproducibility

N/A
